# Are stage-based health information messages effective and good value for money in improving maternal newborn and child health outcomes in India? Protocol for an individually randomized controlled trial

**DOI:** 10.1186/s13063-019-3369-5

**Published:** 2019-05-15

**Authors:** Amnesty LeFevre, Smisha Agarwal, Sara Chamberlain, Kerry Scott, Anna Godfrey, Rakesh Chandra, Aditya Singh, Neha Shah, Diva Dhar, Alain Labrique, Aarushi Bhatnagar, Diwakar Mohan

**Affiliations:** 1 Health Intelligence Initiative, Division of Epidemiology and Biostatistics, School of Public Health and Family Medicine, University of Cape Town, Cape Town, South Africa; 2 Department of International Health, Johns Hopkins Bloomberg School of Public Health, 615 N. Wolfe St, Baltimore, MD USA; 3 BBC Media Action, E-21, Market Lane, Hauz Khas, New Delhi, Delhi 110016 India; 4 BBC Media Action, Ibex House, 42-47 Minories, London, EC3N 1DY England; 5 Oxford Policy Management, 4/6 First Floor, Siri Fort Institutional Area, New Delhi, 110049 India; 6 Bill and Melinda Gates Foundation, Capital Court, Munirka, New Delhi, 110067 India

**Keywords:** Mobile health, mHealth, Maternal messaging, India, Pregnant women

## Abstract

**Background:**

Evidence is limited on the effectiveness of mobile health programs which provide stage-based health information messages to pregnant and postpartum women. Kilkari is an outbound service that delivers weekly, stage-based audio messages about pregnancy, childbirth, and childcare directly to families in 13 states across India on their mobile phones. In this protocol we outline methods for measuring the effectiveness and cost-effectiveness of Kilkari.

**Methods:**

The study is an individually randomized controlled trial (iRCT) with a parallel, partially concurrent, and unblinded design. Five thousand pregnant women will be enrolled from four districts of Madhya Pradesh and randomized to an intervention or control arm. The women in the intervention arm will receive Kilkari messages while the control group will not receive any Kilkari messages as part of the study. Women in both arms will be followed from enrollment in the second and early third trimesters of pregnancy until one year after delivery. Differences in primary outcomes across study arms including early and exclusive breastfeeding and the adoption of modern contraception at 1 year postpartum will be assessed using intention to treat methodology. Surveys will be administered at baseline and endline containing modules on phone ownership, geographical and demographic characteristics, knowledge, practices, respectful maternity care, and coverage for antenatal care, delivery, and postnatal care. In-depth interviews and focus group discussions will be carried out to understand user perceptions of Kilkari, and more broadly, experiences providing phone numbers and personal health information to health care providers. Costs and consequences will be estimated from a societal perspective for the 2018–2019 analytic time horizon.

**Discussion:**

Kilkari is the largest maternal messaging program, in terms of absolute numbers, currently being implemented globally. Evaluations of similar initiatives elsewhere have been small in scale and focused on summative outcomes, presenting limited evidence on individual exposure to content. Drawing upon system-generated data, we explore linkages between successful receipt of calls, user engagement with calls, and reported outcomes. This is the first study of its kind in India and is anticipated to provide the most robust and comprehensive evidence to date on maternal messaging programs globally.

**Trial registration:**

Clinicaltrials.gov, 90075552, NCT03576157. Registered on 22 June 2018.

**Electronic supplementary material:**

The online version of this article (10.1186/s13063-019-3369-5) contains supplementary material, which is available to authorized users.

## Background

Despite the proliferation of mobile health (mHealth) programs, evidence on their effectiveness is limited [1, 2]. mHealth solutions which provide stage-based health information messages to pregnant and postpartum women have been shown to increase knowledge, as well as the utilization of antenatal care (ANC), skilled birth attendance (SBA), and childhood immunization rates in Malawi and Zanzibar [1, 3, 4]. However, efforts to compare and/or generalize these findings are limited by the paucity of data and the quality of evaluations and evidence reporting. To date, none of the summative evaluations have reported data on exposure—defined as a function of whether users receive content (technological performance) and how they engage with that content (behavioral performance)—thus hindering efforts to understand the casual pathways between program inputs and the summative outcome measures observed [3, 5, 6].

Despite evidence limitations, maternal messaging programs are among the few examples of mHealth programs which have been successfully scaled. In four countries globally, programs exist with over one million beneficiaries: *Aponjon* in Bangladesh, *mMitra* and *Kilkari* in India, *Wazazi Nipendeni* in Tanzania, and *MomConnect* in South Africa [7]. As these programs transition from donor funding to full integration with public sector health services, renewed calls to generate evidence on their effectiveness and value for money are emerging.

In India, the Kilkari program is an outbound service that delivers weekly, stage-based audio messages about pregnancy, childbirth, and childcare directly to families on their mobile phones, starting from the second trimester of pregnancy until the child is one year old. Initiated in Bihar in 2012, Kilkari has been scaled to 13 states across India with support from Ministry of Health and Family Welfare (MOHFW) at the national level, National Health Missions (NHM) at the state level, and an alliance of donors (Bill and Melinda Gates Foundation, US Agency for International Development (USAID), Barr Foundation, and UK Department for International Development (DFID)). In the preceding 33 months, Kilkari has delivered pre-recorded audio content to an estimated 8.3 million users—making it the largest maternal messaging initiative underway globally.

In this protocol paper, we outline proposed efforts to measure the effectiveness and cost-effectiveness of Kilkari through an individually randomized controlled trial (iRCT) in four districts of Madhya Pradesh (MP). To complement summative outcome measures, we describe qualitative research which will aim to understand user perceptions of mobile messaging as well as responses to program content, access to mobile phones, and, more broadly, experiences providing phone numbers and personal health information to health care providers in both MP and Rajasthan. Sub-studies to develop validated phone survey tools for the measurement of essential newborn care practices and respectful maternity care (RMC) during pregnancy and childbirth are described in brief here and greater detail elsewhere. Also, described elsewhere are planned secondary analyses of Kilkari data from 13 states where deployments are also underway which will use big data analytics, including machine learning, to explore data quality and develop predictive algorithms for measuring listening levels.

## Methods

### Hypothesis, aims, and objectives

#### Primary research question

Does exposure to mobile health information messages during pregnancy and postpartum improve infant feeding and family planning practices in MP, India?

#### Primary research hypothesis

Exposure to Kilkari messages on infant feeding will correspond to a 5% increased prevalence in the reported practice of exclusive breastfeeding for infants 0–6 months of age and use of modern contraceptive methods at 1 year postpartum between intervention and control arms. The control arm is expected to have a prevalence of 58% for the exclusive breastfeeding indicator and based on the recent NFHS data for MP.

##### Study aim 1. Generate evidence on the effectiveness of Kilkari in four districts of MP

- Objective 1a. Measure changes in reproductive maternal newborn and child health (RMNCH) knowledge, practices, and care-seeking in the intervention arm compared to the control arm
- Objective 1b. Measure the reported prevalence of disrespect and abuse during pregnancy
- Objective 1c. Develop validated phone survey tools for measuring RMC, essential newborn care, and infant feeding practices
- Objective 1d. Administer a phone survey to measure RMC, as well as reported infant feeding and essential newborn care practices

##### Study aim 2. Identify factors which could impede the uptake of Kilkari and exposure to health information messages

- Objective 2a. Measure reported prevalence of disrespect and abuse among women with a birth outcome in the preceding 1–4 months in four districts of MP
- Objective 2b. Identify barriers/facilitators for pregnancy and birth registration of pregnant women and enrolment of their mobile phone numbers in the Maternal and Child Health Tracking System (MCTS), including to the sharing of personal data and mobile phone numbers in MP and Rajasthan, India

##### Study aim 3. Understand the factors underpinning successful receipt of calls in four districts of MP

- Objective 2a. Determine what proportion of calls are received by Kilkari users
- Objective 2b. Determine predictors for exposure to Kilkari content based on user characteristics, Interactive Voice Response (IVR), and call center records

##### Study aim 4. Determine whether users are listening to calls received in four districts of MP

- Objective 3a. Examine user perceptions of mobile messaging and use of data for program design and decision-making in MP, India
- Objective 3b. Measure exposure to Kilkari content based on successful delivery of calls (technological performance) and user engagement (behavioral performance) in four districts of MP

##### Study aim 5. Determine the value for money of Kilkari in four districts of MP

- Objective 5a. Measure economic costs from a societal perspective to start-up and support ongoing implementation of Kilkari
- Objective 5b. Measure incremental changes in the coverage of services and uptake of recommended RMNCH practices among women exposed to Kilkari messages vs those not exposed in four districts of MP
- Objective 5c. Determine the incremental cost per disability adjusted life years (DALYs) averted of exposure to Kilkari compared to a null scenario of no messages
- Objective 5d. Conduct sensitivity analyses on key drivers of costs and effects

### Intervention description and enrollment

Kilkari is comprised of 90 min of content delivered through 72 once weekly voice calls: 24 during pregnancy, 24 within the first 6 months postpartum, and 24 from 6 to 12 months postpartum. An estimated 16% of content is comprised of health information on family planning, 13% on infant feeding, 11% on child immunizations, 10% on pregnancy care, 8% on child malnutrition, 7% on diarrhea, and 7% on postnatal care (Table 1).

**Table 1:** Messaging duration in seconds by content area

Content area	Duration in seconds (Topic + content)	
Family planning	874	16%
Infant feeding: breastfeeding, complementary feeding	686	13%
Child immunizations	568	11%
Pregnancy care: ANC, rest, birth preparedness, danger signs, Tetanus toxoid	559	10%
Child malnutrition, anemia, growth monitoring	455	8%
Diarrhea	379	7%
Postnatal care: newborn danger signs, cord care, hypothermia	370	7%
Child health, *Rashtriya Bal Swasthya Karyakram* (RBSK)	299	6%
Delivery: institution, pre-term, home, ambulance	297	6%
Maternal nutrition/anemia/IFA	235	4%
Safe drinking/hygiene/hand washing	167	3%
*Janani Shishu Suraksha Karyakaram* (JSSK)	159	3%
Pneumonia	130	2%
Childhood diseases	83	2%
Test for pregnancy	72	1%
Malaria	66	1%
Total messaging time in seconds	5399	100%

In the 13 states where Kilkari implementation is ongoing, pregnant woman or postpartum women’s data are entered into one of two health information systems registries: maternal and child health tracking system (MCTS) or reproductive and child health (RCH) system. Data, including mobile phone numbers, are collected in paper registers by frontline health workers, including auxiliary nurse midwives (ANMs) and accredited social health activists (ASHAs), and entered by data entry operators (DEOs) into the government’s electronic health records system—either MCTS or RCH system depending on the state. Data are then sent from there to the MOTECH system. Before the data are accepted by MOTECH, the system automatically runs validations to check that the mobile numbers are in the correct format, locations match location masters in the MOTECH database, and last menstrual period (LMP) and date of birth are within the Kilkari timeframe. The MOTECH system then determines the schedule for Kilkari messages based on the LMP or delivery date and the MOTECH engine provides the list of phone numbers (clients) to be called each day to the IVR system, which then calls the numbers and plays the appropriate pre-recorded message.

For the purposes of this intervention, mobile numbers will be collected directly by the research team for randomization. Pregnant and postpartum women randomized to receive Kilkari messages will receive messages from their enrollment in pregnancy through to 12 months postpartum. Women enrolled to the comparison arm (status quo) will not receive messages.

### Theory of change

**Fig. 1:**
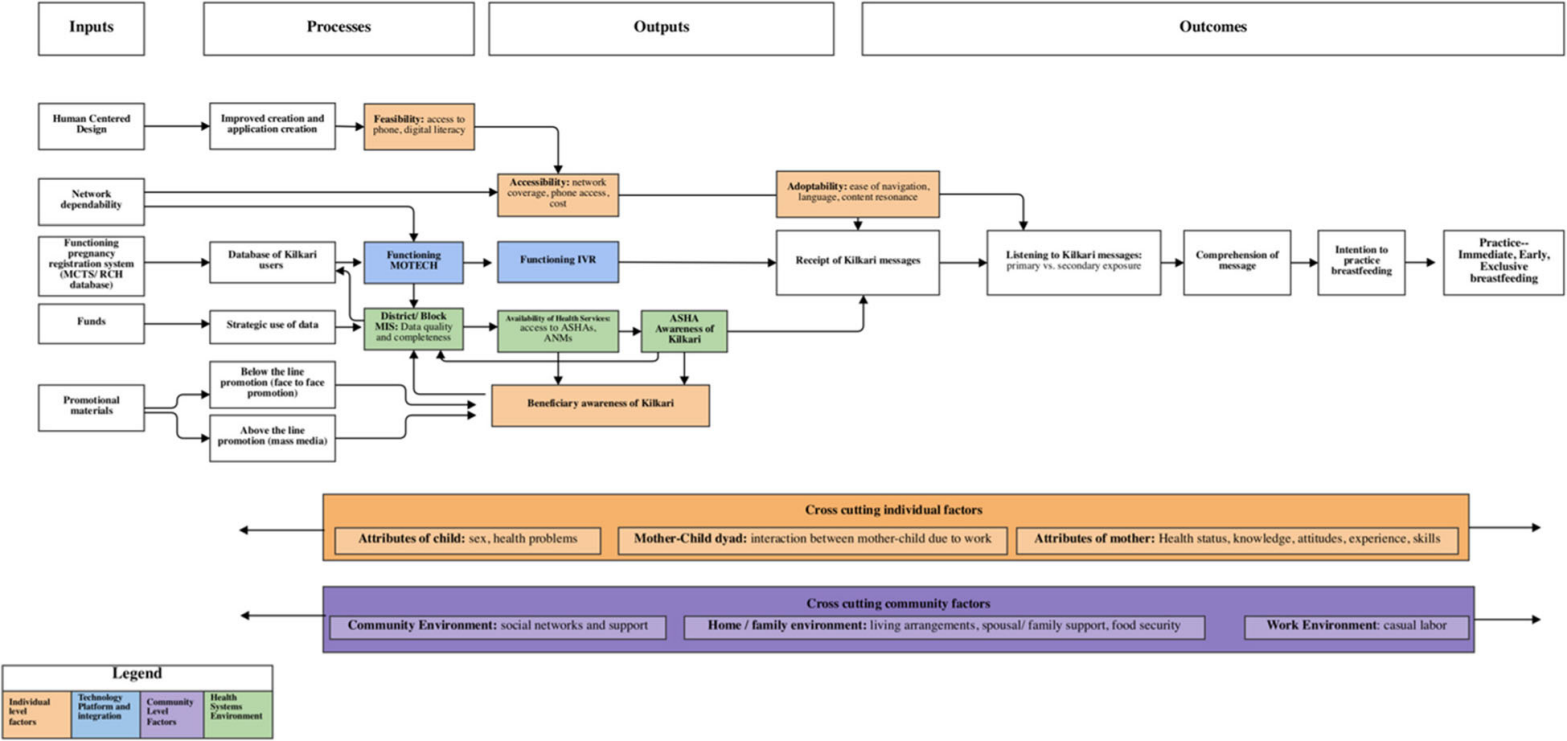
Theory of change for assessing the impact of Kilkari

Figure 1 depicts a theory of change for Kilkari’s impact on health outcomes specific to immediate and exclusive breastfeeding. This theory of change builds off of frameworks proposed by Kimani-Murage et al. [8] and Rollins et al. [9] for assessing factors affecting the actualization of breastfeeding recommendations.

### Study design

The study is an individually randomized controlled trial (iRCT) with a parallel, partially concurrent, and unblinded design.

### Participants and study setting

Study participants will include pregnant and postpartum women from four districts (Rajgarh, Rewa, Hoshangabad, Mandsaur) of MP where Kilkari implementation has not yet occurred. Additional qualitative research specific to MCTS/ RCH will be carried out in two districts (Pali and Sikar) of Rajasthan. In both states, district selection was made by the MOHFW with inputs from the implementing partner BBC Media Action.

The study setting in MP is characterized by disparities in access to education, mobile phones, and health services by gender and geographic location (rural/urban). With a population of over 75 million, MP is home to over 20% of India’s population. MP ranks as one of the worst performing states in India economically (gross domestic product per capita of $1100 USD versus $1709 nationally) and in terms of health outcomes, particularly with regard to child nutrition. In 2015, only 35% of children were breastfed within one hour of birth and 58% of children exclusively breastfed until 6 months [10]. One in four children aged under 5 years were wasted (weight-for-height), and 42% were stunted (height-for-age) [10]. While over half of pregnant women attended ANC in the first trimester, only 36% received the recommended four ANC visits [10]. Rates of institutional delivery were high with over 81% of women reportedly delivering in a health facility in 2015, and yet only 18% received a postnatal health check within 2 days following birth [10]. Health behaviors and care-seeking practices differ markedly between urban/rural areas and are underpinned by high rates of illiteracy (41% of women, 18% of men) and poor access to mobile phones among women [10]. In 2015, 19% of rural and 50% of urban women reported having access to a mobile phone [10].

### Trial enrollment

Trial inclusion will be restricted to women who are 12–34 weeks of gestation at randomization, ≥ 18 years of age, speak and understand Hindi, and own or have access to a mobile phone during the morning and afternoon. Women will be excluded who are BSNL mobile subscribers (due to poor network coverage) and do not provide consent to participate in the trial.

To identify eligible women, all FLHWs in the study villages will be contacted to obtain a list of the currently pregnant women in their village. If the FLHW is not available, a census of those villages will be carried out to find women greater than 18 years of age, within the fifth to seventh month (12–34 weeks of gestation at randomization) of their pregnancy. Women who complete the face to face survey and consent to receive Kilkari messages will be randomized to receive Kilkari calls (intervention) or not receive messages (comparison). Intervention area participants will start to receive Kilkari calls by no later than 8 months (34 weeks) after conception. The timing of this enrolment is intended to mirror that observed in other state deployments of Kilkari which rely on the identification of pregnant women via the MCTS/RCH database and in practice therefore enroll women in the later stages of pregnancy and/or early postpartum.

### Allocation and randomization

Gestational age, parity, age of woman, and ownership of phone are important variables potentially associated with exposure (listening to messages) and are likely to influence outcomes. The sample will be stratified by the above variables to allow for equal numbers of these categories in the sample between intervention and control groups (balance of covariates in the sample). Randomization will be performed using the *sample* command in stata with the use of the above listed variables as stratifiers. The randomization procedure will be done in blocks (block randomization) after the enrollment is completed for each sub-district administrative unit called as Community Development Block. This is to ensure a similar number of intervention and control subjects for each Community Development Block.

### Sampling

Table 2 summarizes the sample size requirements by activity, along with underpinning assumptions. To determine changes in RMNCH knowledge, practices, and careseeking over time (study aim 1), sampling will be powered to detect changes in coverage across study arms. To detect a 5% difference in reported practice of exclusive breastfeeding assuming an alpha of 0.05, 80% power, the estimated sample size for a two-sample proportions test would be 3200 women. After adjusting for a design effect (variance inflation factor) of 1.25 due to clustering at the level of the Community Development Block and a 20% loss to follow-up from enrollment to endline surveys and a potential loss of 35% of women due to poor reporting of phone numbers and/or changes to original phone numbers provided, a total of 5000 women will be enrolled to the study. Assuming 40% of women have access to a mobile phone, we anticipate that nearly 700,000 households will be listed in all four districts of MP.

**Table 2:** Sample size estimates by study aim

Study aim	Survey	Target population	Sample size	Assumptions
Study aim 1a*	Face to face survey to measure changes in infant feeding practices	Women 5–7 months pregnant in four districts of MP	5000	• 5% change in reported practice of immediate and exclusive breastfeeding • Loss to follow-up of 20% from enrolment to final follow-up visit • Variance inflation factor of 1.25
Study aim 1b	Phone survey to measure changes in infant feeding	Postpartum women randomized to Kilkari	5000	Descriptive survey; administered to all women enrolled into the study
Study aim 1c*	Test-retest	Women 5–7 months pregnant in four districts of MP	168 women to be interviewed twice	• Proportion of positive responses of 0.35 for rater 1 and 0.40 for rater 2 • Adjusting for a 15% loss to follow-up/refusal between the first and second surveys
Study aim 1*	Phone survey for postpartum women: infant feeding, essential newborn care, RMC during childbirth	Women 1–4 months postpartum in two districts of MP	880 women interviewed as part of face to face surveys contacted to yield 146 completed face to face and phone survey interviews	• 20% phone survey response rate • Proportion of positive responses of 0.35 for rater 1 and 0.40 for rater 2
Study aim 2*	Phone survey for pregnant women: RMC during pregnancy	Women 5–7 months pregnant in four districts of MP	880 women interviewed as part of face to face surveys contacted to yield 146 completed face to face and phone survey interviews	• 20% phone survey response rate • Proportion of positive responses of 0.35 for rater 1 and 0.40 for rater 2
Study aim 2	MCTS/RCH registration	• FLHWs • Block and district medical officers • Data entry officers	64 in-depth interviews, 24 focus group discussions, and 24 observations	
Study aim 4	User perceptions of Kilkari	• FLHWs: ASHAs, ANMs • Women enrolled to Kilkari and male phone owners	49 in-depth interviews and six focus group discussions	

*Sample estimates based on kappa of 0.80, alpha of 0.05, margin of error of 0.05%

Among pregnant women, a module on RMC during antenatal care will be integrated into the baseline assessment amongst the enrolled women. To measure RMC during childbirth, a total of 880 women with a birth outcome in the preceding 1 to 4 months will be interviewed. This sample size was designed to accommodate inter-modal testing and is sufficient to additionally measure the prevalence of the RMC indicator of reported verbal abuse during childbirth with 80% power, alpha of 0.05, and precision of 5%.

To generate validated phone survey tools for RMC, we will aim to determine the inter-modal reliability of face to face versus computer-assisted telephone interviewing (CATI)/phone surveys for both pregnant and postpartum women. Assuming a kappa of 0.80, a margin of error of 0.05, an alpha of 0.05, and the proportion of positive responses of 0.35 for rater 1 and 0.40 for rater 2, 146 participants who have completed the survey are required. Adjusting for a 20% loss to follow-up between the face to face women’s survey and the following mobile phone survey, and a 20% baseline completion percentage for the latter, 880 women with a birth outcome in the preceding 1 to 4 months will be interviewed face to face. Within 1–2 weeks of the initial interview, a random sample of those completing the face to face interview who consent to be called for the follow-up phone survey will be contacted. Assuming a 20% response rate, all 880 women will be contacted as part of the phone survey to yield the 146 completed face to face and phone survey interviews.

### Measurement

**Fig. 2:**
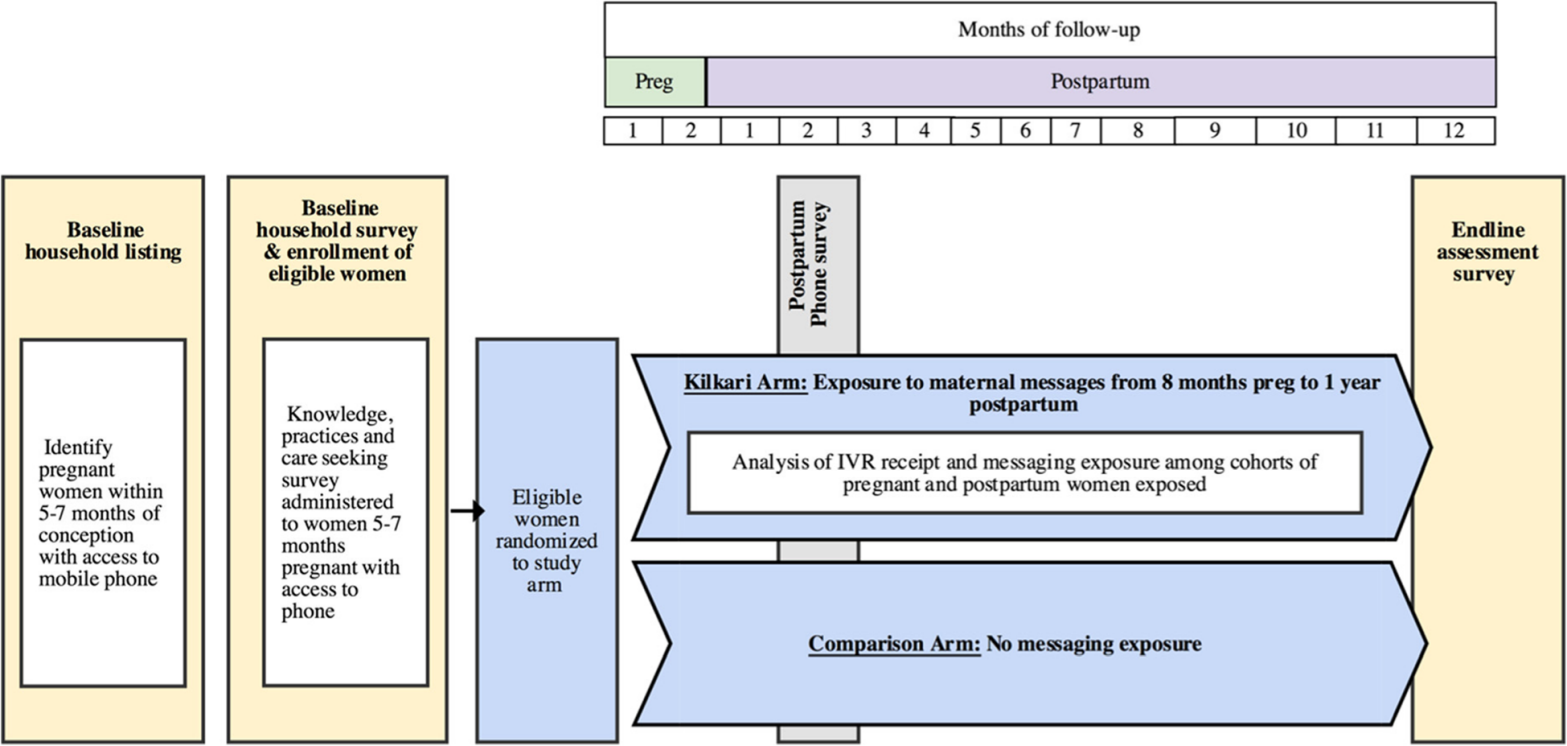
Overview of enrolment and study design over time

**Fig. 3:**
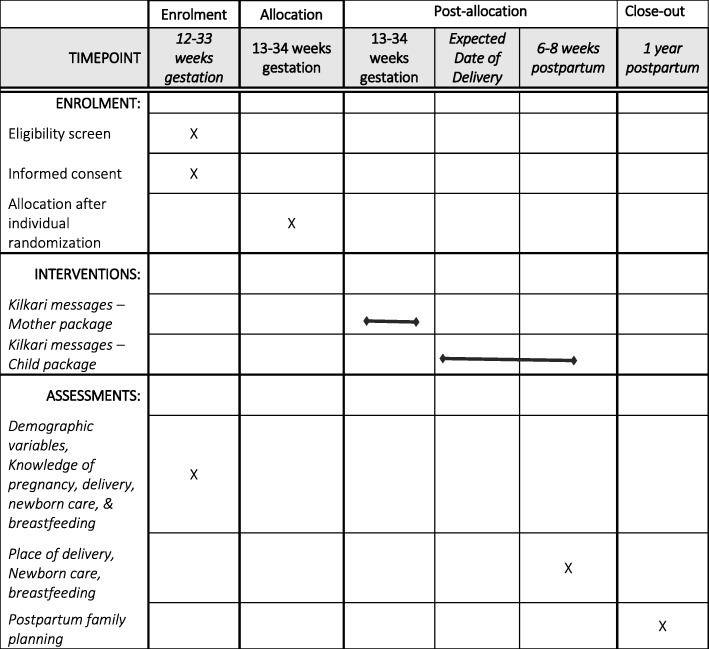
Standard Protocol Items: Recommendations for Interventional Trials (SPIRIT) Checklist

Figure 2 provides an overview of enrolment and study design over time and the Standard Protocol Items: Recommendations for Interventional Trials (SPIRIT) are presented in Fig. 3 and Additional file 1. At enrollment, women will be administered a survey containing modules on phone ownership, geographical and demographic characteristics, knowledge (danger signs during pregnancy, delivery, and postpartum; essential newborn care; nutrition; hygiene, diarrhea treatment, family planning, etc.), practices (immediate, early, and exclusive breastfeeding; complementary feeding; skin-to-skin care, etc.), RMC, and coverage for antenatal care, delivery, and postnatal care. Around 6–8 weeks after the expected date of delivery, a phone survey will be administered to a random sample of the enrolled women until a target sample size of 3500 is attained (1750 each in intervention and control arms) to assess breastfeeding and newborn care practices. An endline survey will be carried out from November 2019 to February 2020 and each woman will be followed up at 12 months postpartum based on their expected date of delivery at enrollment. The endline survey will capture data on practices during pregnancy and intrapartum and postpartum periods.

#### Study aim 1: Generate evidence on the effectiveness of Kilkari in four districts of MP

Knowledge attitudes and practices (KAP) surveys will be administered at baseline and endline containing modules on phone ownership, geographical and demographic characteristics, knowledge (danger signs during pregnancy, delivery, and postpartum; essential newborn care; nutrition; hygiene, diarrhea treatment, family planning, etc.), practices (immediate, early, and exclusive breastfeeding; complementary feeding; skin-to-skin care, etc.), RMC, and coverage for antenatal care, delivery, and postnatal care. Questions on exposure to and sources for health information content will additionally be asked to identify possible sources of contamination. The list of individuals randomized to receive Kilkari content will be provided to BBC Media Action for enrollment into Kilkari continuously throughout the implementation of the baseline survey. Following 11–12 months, participants from both study arms will be interviewed as part of the face to face endline survey.

A phone survey will be administered to all study arm participants on a rolling basis at 1 month postpartum to measure RMNCH practices, including infant feeding practices and essential newborn care as well as RMC. Methods to generate a validated phone survey tool are described elsewhere and in brief will include a face to face survey administered to women during pregnancy and with a live birth outcome in the preceding 1–4 months postpartum at the time of the household listing. A sub-sample of respondents from the initial face to face survey will be administered a second test-retest survey by a separate but similar profile of enumerator. Findings from the face to face surveys will be used to generate phone survey tools which will be implemented amongst a random sample of pregnant and postpartum women who consent to be called for the follow-up phone survey. Analyses to assess the intermodal reliability of face to face versus phone survey responses will be used to generate a validated tool. The tool developed for postpartum women will subsequently be administered to women enrolled into the iRCT during pregnancy at 1–4 months postpartum (Fig. 2).

#### Study aim 2: Identify factors which could impede the uptake of Kilkari and exposure to health information messages

User experiences with RMNCH services received in the public sector, including care received during pregnancy and childbirth, are anticipated to influence decision-making on whether recommended practices promoted through Kilkari messages are adhered to. Women’s experiences with health services during pregnancy (RMC) will be measured as part of the baseline face to face survey administered to women 5–7 months pregnant. In addition to user experiences with RMNCH, in-depth interviews (IDIs), focus group discussions (FGDs), and direct observations will be carried out among pregnant and postpartum women to identify barriers to and facilitators of pregnancy and birth registration as well as the enrolment of their mobile phone numbers in MCTS/ RCH, including the sharing of personal data and mobile phone numbers in MP and Rajasthan, India.

#### Study aim 3: Understand the factors underpinning successful receipt of calls

Following enrollment to Kilkari, we will draw from system-generated data to determine factors underpinning the successful receipt of calls not only among iRCT participants but across the 13 states where Kilkari implementation is underway. Since it is not possible to discern who listens to calls, emphasis will be placed on the mobile phone number registered to receive Kilkari content as the unit of analysis. Predicators for exposure to Kilkari content will be captured by four stakeholders: 1) government’s data system (MCTS, RCH, webservices); 2) program’s call delivery and receipt systems; 3) mobile network operator (network coverage and quality); and 4) user device characteristics (switched on, within range of network). Table 3 summarizes key indicators proposed for assessment.

**Table 3:** Essential steps and barriers to exposure to Kilkari content in four districts of MP

	Steps in implementing Kilkari	Critical challenges	Illustrative indicators
Critical points of drop-out for Kilkari users	Pregnancy is registered on MCTS/RCH database	• Data quality, including phone numbers provided* • Delays in pregnancy identification*	Percentage of pregnant women in the MCTS/RCH with a phone number Percentage of pregnant women in the MCTS/RCH with an active phone number
	Pregnant woman becomes eligible to start receiving Kilkari messages	• Delays in registration and transfer of data across districts	Days between pregnancy identification and date of first Kilkari call
	Pregnant woman receives calls	• Variations in network coverage • Phone number may not be active • Phone number may not belong to a relevant woman or a family that contains a relevant woman—may just be a random number • Woman may not be the primary owner of the phone • Woman may not have access to phone • Phone may not be charged	Percentage of calls received out of those intended per mobile device registered to Kilkari Leading reasons for call failure
	Pregnant woman listened to the calls	• User engagement with content • User access to the phone • User mobile literacy • Phone functionality, including charge	Percentage of calls listened to out of those received on average among those enrolled to Kilkari Percentage of calls listened to on average out of all calls received Days and times during the day when messages are most likely to be listened to completely Percentage of mobile phones who listened to 75–100% of the calls on average among all phone numbers enrolled to Kilkari
	Pregnant women continues engaging with Kilkari	• Phone number may not be active • Woman may grow disinterested in continuing the service	Percentage of mobile phones with answering all weekly calls each month (4–5 depending on the month and their subscription data) Percentage of phones who received and listened to the entire Kilkari package among all phone numbers randomized to receive messages

*Less relevant to iRCT as eligible users will be identified as part of the household listing at baseline and a list of phone numbers provided to BBC Media Action for content delivery

#### Study aim 4: Determine whether users are listening to calls received

To measure user engagement with Kilkari content (behavioral performance), we will measure the proportion of calls listened to out of those received on average among the mobile phones enrolled to Kilkari. MOTECH data will be merged with IVR, call center data, and baseline face to face survey data on user characteristics, including socioeconomic status, education, phone access, mobile literacy, and numeracy, and used to identify predictors for exposure to Kilkari content. Differentials in listening patterns for each mobile device will be used to classify Kilkari users into high and low listenership groups. In addition to quantitative data analyses, qualitative interviews will be carried out to examine user perceptions of mobile messaging and use of data for program design and decision-making. Differentials in user perceptions of content by topical area as well as over time will be assessed to facilitate understanding of possible differentials in user engagement.

#### Study aim 5: Determine the value for money of Kilkari in four districts of MP

To measure the economic costs of Kilkari we will prospectively measure program, user, and health system costs in the four districts of MP. Program costs associated with the development, start-up, and implementation of program activities will be assessed using an ingredients approach for the 2018–2019 analytic time horizon. Costs to users will be assessed through face to face household surveys and include direct (out of pocket costs for drugs, supplies, consultation fees, transportation) and indirect (wages lost, child care) costs incurred by users for RMNCH careseeking. Finally, we will explore the feasibility of measuring incremental costs to the health system above and beyond those captured by the measurement of program and user costs. Collective consideration of program, user, and health system costs will allow for a societal perspective to be taken and thus a more comprehensive estimate of the full economic costs associated with the program across study arms of individuals exposed versus those not exposed to Kilkari. To measure the incremental effectiveness of Kilkari, data on changes in coverage for RMNCH careseeking and practices assessed through household surveys will be inputted into the Lives Saved Tool to generate a measure of lives saved associated with program activities. Lives saved will be used to yield an estimate of the incremental DALYs averted and ultimately a cost per DALY averted for Kilkari.

### Analyses

The statistical analysis plan will ensure that the analyses are not data driven or selectively reported. The presentation of primary analyses is expected in spring 2020, after all participants have been followed up to 1 year postpartum. Unadjusted and adjusted results will be presented as appropriate effects sizes with 95% confidence intervals (CI). Demographic and population characteristics of the women at baseline will be compared between study groups. Significance tests will be performed to investigate for differences between groups at baseline. Categorical outcomes will be analyzed using contingency tables and chi-square test. Continuous normally distributed variables will be analyzed using *t*-tests and non-normally distributed variables using non-parametric tests. The primary outcomes, early and exclusive breastfeeding and the adoption of modern contraception at 1 year postpartum, will be analyzed by intention-to-treat methodology, with adjustment for stratifying and baseline factors, including gestational age at enrollment, ownership of mobile phone, age of the pregnant woman at enrollment, and birth order of index pregnancy. Binary logistic regression (BLR) models with generalized estimation equations will be used to account for clustering effects at the level of the Community Development Block. The secondary outcomes of interest are anticipated to include immediate breastfeeding, skilled attendance and facility delivery, postnatal care, newborn birth practices, and immunizations. Binary logistic regression will be used for binary outcomes, and multiple linear regression will be used for continuous measures. Wilcoxon rank sum test will be used for continuous measures which are not normally distributed, including duration of breastfeeding. Cox proportional hazards regression will be used for time to event analyses, including time to initiation of breastfeeding and adoption of a modern contraceptive method in the postpartum period. All regression analyses will be performed with adjustment for baseline factors.

Comparisons between the treatments will be performed in pre-specified subgroups, including phone ownership, caste, age groups, etc. The subgroup analysis does not comprise the primary analysis and did not inform the sample size calculation. The interpretation of any heterogeneity in effects due to the subgroups will be based on using interaction terms between the sub-group variables and the intervention group with no adjustment for multiple testing. The exposure of women to different messages will be assessed using the system-generated data from the MOTECH database. We will analyze the effect of different messages on specific related outcomes using a per-protocol methodology. Missing data will be assessed for any systematic biases based on the dropout of women using their baseline characteristics. Sensitivity analysis will be performed to assess the effect of different levels of systematic bias on the effect sizes.

#### Phone surveys

The phone survey analyses will principally aim to determine the reliability of the phone survey tools. Reliability analyses will aim to determine the stability and consistency of results [11]. A Cronbach’s alpha of 0.7 or higher is proposed as the cutoff for determining sufficient evidence of reliability [11]. Additional analyses related to the internal consistency of the scale as well as the presence of floor and ceiling effects will be conducted and overall findings summarized in an overarching table on validity and reliability [11].

#### Economic evaluation

A full health-economic analysis will only be performed after completion of the main trial. Once costs and effects are estimated, uncertainty analyses, including one-way and probabilistic sensitivity analyses, will be conducted. The latter will be carried out in Microsoft excel using a Monte Carlo simulation with 1000 iterations per analysis. The resulting mean point estimate will be obtained by dividing mean costs by mean effects, while a 95% confidence interval for the ICER is presented based on percentiles. A cost effectiveness plane and cost effectiveness acceptability curve will be used to calculate the probability that the intervention will be cost-effective for each of a number of standard thresholds of cost-effectiveness. Cost-effectiveness will ultimately be determined according to thresholds set forth by the Commission for Macroeconomics and Health and WHO which stipulate that an intervention is “highly cost-effective” and “cost-effective” interventions at one and three times, respectively, the value of per capita gross domestic product per DALY averted. To facilitate comparison with alternative resource uses, we will additionally compare findings against those available in the literature.

## Discussion

This is the first study of its kind in India to determine the effectiveness and value for money of a maternal mobile messaging initiative. The iRCT proposed aims to ensure rigor in the generation of evidence by linking exposure to content assessed to summative outcome measures, including family planning, essential newborn care, and infant feeding practices. Efforts to measure exposure will draw upon system-generated data to consider both the technological (successful receipt of calls) and behavioral performance (user engagement with calls received) of the program. Qualitative research will complement these efforts by facilitating understanding of barriers to and facilitators for the provision of phone numbers to public sector providers (which is integral to individual entry to the program) as well as user engagement with and perceptions of content. Additional data collected on RMC will seek to better understand how women’s experiences with careseeking during pregnancy and childbirth may influence the uptake of Kilkari content. Finally, cost-effectiveness analyses will aim to compare incremental costs and consequences of exposure to Kilkari versus status quo of no exposure in the four districts of MP where the iRCT is being implemented. Collectively, this portfolio of research activities aims to provide the strongest evidence to date on maternal messaging in India.

Elsewhere, such as in Zanzibar and Malawi, maternal messaging initiatives have demonstrated a significant effect on utilization of health services and health outcomes. In Zanzibar, the Wired Mothers Program provided unidirectional text messaging and direct two-way communication using a free call voucher system to provide reminders for ANC visits, stage-based RMNCH information, and an emergency medical response system. Program activities were associated with an increase in four or more ANC (odds ratio (OR) 2.39, 95% confidence interval (CI) 1.03 to 5.55) [3], in SBA among urban women (OR 5.73, 95% 1.51–21.81) [5], and reduced perinatal mortality (OR 0.50, 95% CI 0.27 to 0.90) [4]. In Malawi, the Chipatala Cha Pa Foni program used a toll-free hotline to provide health information and advice, as well as tips and reminders by mobile messaging tailored to the client’s week of pregnancy and/or child’s age. Program activities were associated with improved RMNCH knowledge and behavior, including increased utilization of ANC within the first trimester, increased bed-net use for pregnant women and children, and breastfeeding within one hour of birth [12]. Since these findings were published, the Ministry of Health and Population-Malawi announced efforts to scale Chipatala Cha Pa Foni from nine to all 28 districts by the end of 2018. While both of these programs provide a strong foundation of evidence, variations in population and health system characteristics, scale of implementation, as well as program content and delivery strategies, including additional interventions provided concurrently, arguably limit the generalizability of findings.

Despite calls to address broader evidence gaps in linking digital technologies to outcome and impact level health indicators, little to no attention has been paid to measuring critical processes pertaining to the technological and behavioral performance of the program. In the case of Kilkari, efforts to assess the technological performance will aim to identify factors underpinning the successful receipt of calls. In addition to the methods outlined in the preceding sections, we describe proposed applications of machine learning in detail elsewhere, including the development of a classifier for measuring listening levels. In Ghana [4] we previously sought to understand the performance of mobile midwife—a mobile messaging service implemented as part of the Mobile Technology for Community Health (MOTECH) program. Interactive voice response (IVR) message delivery trends suggested that 25% or less of expected mobile health information messages were received by pregnant women [4]. While 20% delivery rates of successful out-bound dialing calls is standard in the mobile industry, improved performance monitoring might have led to the earlier identification of delivery deficits and, in turn, the implementation of strategies which might have improved exposure to content, including call retry logics and systems.

These findings reinforce the need to collect evidence not only on summative evaluation findings, but also on the processes which underpin them. In the case of MOTECH, missing data prevented the measurement of outcome level indicators. Hypothetically, however, had we been able to measure these and assumed target constituents received and listened to messages without measuring exposure, we likely would not have seen an effect and thus concluded that exposure to maternal messages does not influence behavior in the study setting of Ghana. In reality, while it is likely that limited exposure to the program due to technological limitations in message delivery would have eroded any chance for outcome level metrics to be attained, we do not have enough evidence to draw conclusions beyond those highlighting limitations in program reach and exposure. The advantage in having digital health data is that we *can* measure exposure at an individual level and thus if designed appropriately link that with outcome level data. We propose employing the same strategy for assessing Kilkari and thus collecting data on the underlying technological and behavioral performance of the program. We hope that this approach promotes a new standard with regard to linking data on individual level exposure to health outcomes.

### Limitations

As part of this study, the evaluation team will facilitate the identification and enrollment of pregnant women to Kilkari. The timing of this enrollment is intended to mirror that observed in other state deployments of Kilkari which rely on the identification of pregnant women via the MCTS/ RCH health information system databases, and in practice, therefore, enroll women in the later stages of pregnancy and/or early postpartum. By facilitating the enrollment of women directly into Kilkari, we anticipate having significantly higher population level coverage than the program would otherwise garner had enrollment relied on women identified via routine health information systems maintained by public sector providers. In addition, user listening patterns among trial participants may differ from those observed amongst Kilkari users in other states given that trial enrollment occurred through a face to face interaction—a factor which may lead to greater rapport building and in turn improved user engagement with the program. The question of generalizability is an issue to contend with since the study takes great efforts to provide correct phone numbers for the enrolled women, unlike the usual MCTS/RCH registration system. Higher population level coverage could lead to differences in the characteristics of women enrolled in the study compared to those enrolled through routine health information systems, resulting in selection bias. The measurement of outcomes is based completely on self-reports as part of household surveys. This can result in information bias due to recall issues and a social desirability to report ‘appropriate’ behavior and practice. The use of a RCT design is expected to limit the role of confounders but cannot be entirely ruled out.

## Conclusions

Study findings are anticipated to provide the strongest evidence to date on the effectiveness and cost-effectiveness of maternal mobile messaging initiatives in India. Findings will be instrumental for informing future directionality of maternal messaging initiatives in India and elsewhere globally.

## Additional file

Additional file 1: SPIRIT 2013 checklist: Recommended items to address in a clinical trial protocol and related documents*. (DOC 121 kb)

## Abbreviations

ANC: Antenatal care
BMGF: Bill and Melinda Gates Foundation
CATI: Computer-assisted telephone interviews
DALYs: Disability adjusted life years
DFID: Department for International Development
EAs: Enumeration areas
FLHWs: Frontline health workers
iRCT: Individually randomized controlled trial
KAP: Knowledge attitudes and practices
MCTS: Maternal Child Tracking System
mHealth: Mobile health
MOHFW: Ministry of Health and Family Welfare
MOTECH: Mobile Technology for Community Health
MP: Madhya Pradesh
NFHS: National Family Health Survey
RCH: Reproductive child health
RMC: Respectful maternity care
RMNCH: Reproductive, maternal, newborn and child health
SBA: Skilled birth attendance
SPIRIT: Standard Protocol Items: Recommendations for Interventional Trials
